# Sperm Retrieval in Patients with Klinefelter Syndrome: A Skewed Regression Model Analysis

**DOI:** 10.22074/ijfs.2017.4702

**Published:** 2017-02-16

**Authors:** Mohammad Chehrazi, Abbas Rahimiforoushani, Marjan Sabbaghian, Keramat Nourijelyani, Mohammad Ali Sadighi Gilani, Mostafa Hoseini, Samira Vesali, Mehdi Yaseri, Ahad Alizadeh, Kazem Mohammad, Reza Omani Samani

**Affiliations:** 1 Department of Epidemiology and Reproductive Health, Reproductive Epidemiology Research Center, Royan Institute for Reproductive Biomedicine, ACECR, Tehran, Iran; 2 Department of Epidemiology and Biostatistics, School of Public Health, Tehran University of Medical Sciences, Tehran, Iran; 3 Department of Andrology, Reproductive Biomedicine Research Center, Royan Institute for Reproductive Biomedicine, ACECR, Tehran, Iran; 4 Department of Urology, Shariati Hospital, Tehran University of Medical Sciences, Tehran, Iran; 5 Student Research Committee, Mazandaran University of Medical Science, Sari, Iran

**Keywords:** Klinefelter Syndrome, Sperm Retrieval, Logistic Regression

## Abstract

**Background:**

The most common chromosomal abnormality due to non-obstructive azoospermia (NOA) is Klinefelter syndrome (KS) which occurs in 1-1.72 out of 500-1000 male infants. The probability of retrieving sperm as the outcome could be asymmetrically different between patients with and without KS, therefore logistic regression analysis is not a well-qualified test for this type of data. This study has been designed to evaluate skewed regression model analysis for data collected from microsurgical testicular sperm extraction (micro-TESE) among azoospermic patients with and without non-mosaic KS syndrome.

**Materials and Methods:**

This cohort study compared the micro-TESE outcome between 134 men with classic KS and 537 men with NOA and normal karyotype who were referred to Royan Institute between 2009 and 2011. In addition to our main outcome, which was sperm retrieval, we also used logistic and skewed regression analyses to compare the following demographic and hormonal factors: age, level of follicle stimulating hormone (FSH), luteinizing hormone (LH), and testosterone between the two groups.

**Results:**

A comparison of the micro-TESE between the KS and control groups showed a success rate of 28.4% (38/134) for the KS group and 22.2% (119/537) for the control group. In the KS group, a significantly difference (P<0.001) existed between testosterone levels for the successful sperm retrieval group (3.4 ± 0.48 mg/mL) compared to the unsuccessful sperm retrieval group (2.33 ± 0.23 mg/mL). The index for quasi Akaike information criterion (QAIC) had a goodness of fit of 74 for the skewed model which was lower than logistic regression (QAIC=85).

**Conclusion:**

According to the results, skewed regression is more efficient in estimating sperm retrieval success when the data from patients with KS are analyzed. This finding should be investigated by conducting additional studies with different data structures.

## Introduction

The most common chromosomal abnormality due to non-obstructive azoospermia (NOA) is Klinefelter syndrome (KS) which occurs in 1-1.72 out of 500-1000 male infants (1-3). This genetic abnormality can develop meiotic nondisjunction due to a 47,XXY genotype in the majority of cases (4). KS is characterized by infertility, elevated luteinizing hormone (LH) levels, elevated follicle stimulating hormone (FSH), normal or reduced testosterone, normal or increased height, muscle weakness and reduced strength, increased facial hair, osteoporosis, obesity, increased thromboembolic risk, dyslipidemia, and low glucose tolerance. In addition there are slight deficits in very specific domains of cognition, but without an increase in the occurrence of mental retardation (5). Although the typical karyotype is 47,XXY, chromosomal mosaics are specified with 46XY/47,XXY and complements with multiple X chromosomes such as 48XXXY.

It is important to evaluate the chances for sperm retrieval among KS patients and compare them to azoospermia with normal karyotype by using a model which provides more efficient results for researchers. When researchers have more efficient results they can choose the best method to handle this problem and patients may not need to undergo invasive surgeries (6). On the other hand, it can also increase the chances for success and decrease patient expenses. Logistic and probit regressions have already been applied to analyze binary outcome data. The models are fit using a symmetric distribution function. However, the use of such distributions may give biased results in some cases. The purpose of this study is to apply the skewed regression model, as a new regression model, (7) to analyze data collected by microsurgical testicular sperm extraction (micro-TESE) among azoospermic patients with and without non-mosaic KS syndrome who referred to Royan Institute due to infertility. This type of regression has a further parameter, the skewness parameter, which increases its flexibility. This parameter controls the skewness of the link function. Finally, we have compared results from the two models (logistic regression and skewed regression) according to statistical criteria.

## Materials and Methods

This cohort study enrolled 134 patients with KS and 537 patients without KS. Patients were referred to Royan Institute, a referral infertility clinic in Tehran, Iran between 2009 and 2011. We individually matched patients and controls according to disease duration, time of surgery, and surgeon in order to avoid confounding factors such as surgical skills and age.

### Clinical evaluation

The 47,XXY karyotype was confirmed by Gbanding of more than 30 peripheral blood lymphocytes. Semen analysis was performed according to World Health Organization (WHO) guidelines to evaluate sperm parameters. At least two analyses confirmed azoospermia in each patient. Blood samples were taken in the morning to measure FSH, LH, and testosterone levels.

### Microsurgical testicular sperm extraction

Microsurgical testicular sperm extraction (Micro-TESE) was performed on the patients under general anesthesia as described by Schlegel (8). The procedure was considered successful when sperm were retrieved from the patients.

### Statistical modeling

#### Independent binary regression model

The binary logistic regression model, as previously introduced (9), is appropriate for independent outcomes. When y_1_, y_2_, …,y_n_ are a set of n observations of binaryoutcomes such as success and failure in sperm retrieval, which are independent of each other; p_i is the probability of success forpatient “i”; x_1_, x_2_,…, x_n_ are independent predictors; and β_1_, β_2_, …, β_k_ are equal to regression coefficients, then, the binary regression model can be written as follows:

(Model 1) $$p_{i}=F(x_{i}\mathrm{'\beta})$$i=1, …, n

where: F(.) is acumulative distribution function. If F is the distribution function of normal and logistic, then the above model will be alogistic and probit regression model, respectively.

#### Correlated binary regression model with a symmetric link

Assume that a binary outcome “Y_it_" (t=1,^2^,e T) is measured for a particular person during time T. As the outcomes for one person are correlated, in order toevaluate the relationship betweenindependent variables and theoutcome, the earlier model is no longer appropriate. There are different methods to model a relationship between a correlated binary outcome and a set of independent variables. In this study, we have focused on a mixed generalized linear model with a simplified form as follows:

(Model 2) $$p_{\mathrm{it}}=F(x_{\mathrm{it}}\mathrm{'\beta}+b_{i})$$

where b_i is a random effect with normal distribution in this formula. Adding this term into model 1 enables the observations to be considered independently. In model 2, which is similar to the independent model, symmetrical logistic or normal distribution functions are assumed for F.

#### Correlated binary regression model with an asymmetric link

Asymmetric links are used for regression models to enable better data fit. In this study, the dependent regression model as previously introduced by Chen et al. (7) is explained. This model uses a hidden variable structure, which has been introduced by Albert and Chib (10). Binary outcome can be created by a desired cut-off point as zero on a continuous latent variable with a mixed structure. The hidden variables can have distributions such as the logistic or normal. Symmetric or a symmetric distributions can be produced for the variable through a mixed structure. Various asymmetric links also can be produced in this way. The most important property of Chen’s model is that it considers the problem of “skewness”, which controls amount of skewness of a link. The link will be symmetric if skewness is zero.

Data were analyzed by R software (version 3.2.2) using the BB package and GEE pack for asymmetric and symmetric models, respectively. The logistic regression model with the symmetric and asymmetric link was fit to the data and we compared the goodness of fit of the two models by quasi Akaike’s information criteria (QAIC) (11). The level of 0.05 was considered significant.

## Results

The mean age ± SD of patients with KS was 32.64 ± 0.64 years (range: 22-49) and the mean age of participants without the KS was 34.11 ± 0.27 years. The mean serum level for FSH was 34.5 mlU/m Lin KS patients and 22.6 mlU/mL in those without KS. Patients with KS had a mean serum testosterone level of 2.65 ng/mL; those without KS had a mean testosterone level of 4.04 ng/mL. Of 134 patients with KS, 38 patients had sperm retrieved; out of 537 participants, 119 had sperm retrieved. The sperm retrieval rate was 28.4 in KS patients and 22.2 in patients without KS (Table 1).

We performed a marginal effect logistic regression analysis with serum FSH, LH, testosterone, age, and patient groups to determine an association between the probability of sperm retrieval and the covariates during micro-TESE. Adjusted association from the model showed that probability of retrieving sperm during micro-TESE did not differ between the two groups (control and KS) after adjusting for the covariates and could not be predicted by any of the variables. On the other hand, the results obtained from skewed logistic regression showed that the probability of retrieving sperm was not the same for patients with and without KS (P=0.01). QAIC for logistic regression with the symmetric link was equal to 85, whereas for an asymmetric link it was 74. The results of comparing the regression model are shown in Table 2. The proportion of sperm retrieval was almost equal between patients with and without KS.

**Table 1:** Clinical characteristics of patients and controls subdivided according to Micro-TESE outcome


Factor	Klinefelter syndrome			Control		
	Total	Success	Failure	Total	Success	Failure

Age	32.64^a^ ± 0.48	30.0^b^ ± 0.65	33.68 ± 0.6	34.11 ± 0.27	34.6 ± 0.55	33.93 ± 32
FSH (mIU/ml)	34.52^a^ ± 1.4	34.69 ± 2.52	34.44 ± 1.68	22.6 ± 0.83	23.54 ± 1.65	22.22 ± 0.96
LH (mIU/ml)	17.89^a^ ± 1.34	17.0 ± 1.94	18.28 ± 1.24	8.83 ± 0.41	9.22 ± 0.85	8.67 ± 0.47
T (ng/ml)	2.65 ± 0.22	3.4^b^ ± 0.48	2.33 ± 0.23	4.04 ± 0.51	3.52 ± 0.29	4.2 ± 0.67


FSH; Follicle-stimulating hormone, LH; Luteinizing hormone, T; Testosterone, ^a^; Significant compared to the control group, and ^b^; Significant compared with failures in the Klinefelter syndrome (KS) group. Data are presented as mean ± SD.

**Table 2:** Results of logistic regression and skewed regression comparing sperm retrieval between KS and control groups after adjusting for LH, FSH, age and testosterone


Parameter	Logistic regression^a^ (95% CI)	P value	Skewed regression^a^ (95% CI)	P value

Group				
Control	Reference		Reference	
Klinefelter	0.35 (-0.92 _ 0.21)	0.22	-0.49 (-0.79 _ -0.19)	0.01
LH (mIU/mL)	-0.01 (-0.04 _ 0.02)	0.51	-0.02 (-0.03 _ 0.01)	0.26
FSH (mIU/mL)	0.01 (-0.01 _ 0.03)	0.34	0.03 (-0.01 _ 0.07)	0.28
Testosterone (ng/mL)	-0.001 (-0.03 _ 0.03)	0.95	-0.002 (-0.03 _ 0.01)	0.48
Age	-0.01 (-0.05 _ 0.03)	0.66	-0.01 (-0.04 _ -0.02)	0. 4


FSH; Follicle-stimulating hormone, LH; Luteinizing hormone, CI; Confidence interval, a; Significant compared to the control group, and b; Significant compared with failures in the Klinefelter syndrome (KS) group.

## Discussion

The present study has compared sperm retrieval in azoospermia patients with and without KS by two regression models. Sperm from KS patients is typically retrieved by conventional TESE and micro-TESE techniques. Although both techniques have successfully retrieved sperm in 44% of patients, a comparison between conventional TESE and micro-TESE showed that micro-TESE had a higher success rate (55%) compared to conventional TESE (44%) (12). Sperm retrieval rate in men with KS by micro-TESE ranged from approximately 21-72% (13-19). A study by Madureira et al. (20) reported a sperm recovery rate of 38.5% among non-mosaic KS patients. Possibly the rate of sperm retrieval in the current non-mosaic KS series (28.4%) could be compared with those previously reported. A pilot study proposed that sperm retrieval rates in adolescents with KS could be compared with those reported in older men (21).

In some studies it was found that the sperm recovery rate in men with KS reduced with increased age, however there were no effects on serum FSH, LH, or testosterone levels (22-25). Medical and ethical issues were mentioned related to sperm retrieval in adolescent males with KS in a study by Okada et al. (26). Whether previous testosterone treatment, could be considered or not be as a barrier in the sperm retrieval. In our study, skewed logistic regression showed that only younger men had a higher sperm recovery rate compared to older men which suggested that aging could reduce successful sperm recovery in men with KS. In the KS group, a comparison of laboratory parameters between men with successful sperm retrieval and those with failed sperm retrieval showed a significantly higher testosterone level in patients with successful sperm retrieval (3.4 ± 0.48 ng/ml) compared to those with failed sperm retrieval (2.33 ± 0.23 ng/ml). In patients without KS no difference existed between age, FSH, LH, and testosterone levels in patients who experienced successful sperm retrieval compared to those with failed sperm retrieval. Our study reported higher sperm retrieval rates in patients with KS compared to the control group, which could be related to confounder distribution in the two groups and their associations with the outcome. The skewed regression was more powerful than logistic regression to detect this relation. This finding differed from previous studies (19, 22, 23). This result has shown that KS patients can be hopeful for sperm detection and subsequent pregnancy outcomes.

We used logistic regression with symmetric and asymmetric links for data analysis. To the best of our knowledge, this was the first study that applied a regression model with asymmetric link in reproduction research and in this group of infertile patients. The incorrect use of asymmetric link instead of an asymmetric link could lead to a poor fit and result in biased estimates of the regression coefficients. Chen’s model that has been used in this study was more flexible than common models. Therefore, the QAIC value was lower and has shown a better fit than logistic regression with a symmetric link (27, 28). In addition, sometimes it is not possible to control all confounding factors. Hence, the confounding effects should be adjusted by regression model. Thus, a better fit model can provide a more accurate result. The importance of selecting a more appropriate model is specified in such studies more than ever.

## Conclusion

Our findings have revealed that the logistic regression model with an asymmetric link is more flexible and a better fit than the conventional regression model. Since this is the first time a skewed regression has modeled data from infertility studies, we recommend that additional studies and analyses be conducted to evaluate how well this model fits a set of observations. It is also should be notice, sperm could be retrieve of non-mosaic KS as well as without KS patients.

## References

[1] Aksglaede L, Wikström AM, Rajpert-De Meyts E, Dunkel L, Skakkebaek NE, Juul A (2006). Natural history of seminiferous tubule degeneration in Klinefelter syndrome. Hum Reprod Update.

[2] Foresta C, Galeazzi C, Bettella A, Marin P, Rossato M, Garolla A (1999). Analysis of meiosis in intratesticular germ cells from subjects affected by classic Klinefelter's syndrome. J Clin Endocrinol Metab.

[3] Morris JK, Alberman E, Scott C, Jacobs P (2008). Is the prevalence of Klinefelter syndrome increasing?. Eur J Hum Genet.

[4] Harari O, Bourne H, Baker G, Gronow M, Johnston I (1995). High fertilization rate with intracytoplasmic sperm injection in mosaic Klinefelter's syndrome. Fertil Steril.

[5] Höckner M, Pinggera GM, Günther B, Sergi C, Fauth C, Erdel M (2008). Unravelling the parental origin and mechanism of formation of the 47,XY,i(1)(q10) Klinefelter karyotype variant. Fertil Steril.

[6] Zang KD, Bandmann HJ, Breit R, Perwein E (1984). Genetics and cytogenetics of Klinefelter’s syndrome. Klinefelter’s syndrome.

[7] Chen MH, Dey DK, Shao QM (1999). A new skewed link model for dichotomous quantal response data. J Am Stat Assoc.

[8] Schlegel PN (1999). Testicular sperm extraction: microdissection improves sperm yield with minimal tissue excision. Hum Reprod.

[9] Agresti A, Kateri M (2011). Categorical data analysis.

[10] Albert JH, Chib S (1993). Bayesian analysis of binary and polychotomous response data. J Am Stat Assoc.

[11] Pan W (2001). Akaike's information criterion in generalized estimating equations. Biometrics.

[12] Fullerton G, Hamilton M, Maheshwari A (2010). Should non-mosaic Klinefelter syndrome men be labelled as infertile in 2009?. Hum Reprod.

[13] Levron J, Aviram-Goldring A, Madgar I, Raviv G, Barkai G, Dor J (2000). Sperm chromosome analysis and outcome of IVF in patients with non-mosaic Klinefelter's syndrome. Fertil Steril.

[14] Westlander G, Ekerhovd E, Granberg S, Hanson L, Hanson C, Bergh C (2001). Testicular ultrasonography and extended chromosome analysis in men with nonmosaic Klinefelter syndrome: a prospective study of possible predictive factors for successful sperm recovery. Fertil Steril.

[15] Madgar I, Dor J, Weissenberg R, Raviv G, Menashe Y, Levron J (2002). Prognostic value of the clinical and laboratory evaluation in patients with nonmosaic Klinefelter syndrome who are receiving assisted reproductive therapy. Fertil Steril.

[16] Vernaeve V, Staessen C, Verheyen G, Van Steirteghem A, Devroey P, Tournaye H (2004). Can biological or clinical parameters predict testicular sperm recovery in 47,XXY Klinefelter's syndrome patients?. Hum Reprod.

[17] Schiff JD, Palermo GD, Veeck LL, Goldstein M, Rosenwaks Z, Schlegel PN (2005). Success of testicular sperm extraction [corrected] and intracytoplasmic sperm injection in men with Klinefelter syndrome. J Clin Endocrinol Metab.

[18] Koga M, Tsujimura A, Takeyama M, Kiuchi H, Takao T, Miyagawa Y (2007). Clinical comparison of successful and failed microdissection testicular sperm extraction in patients with nonmosaic Klinefelter syndrome. Urology.

[19] Yarali H, Polat M, Bozdag G, Gunel M, Alpas I, Esinler I (2009). TESE-ICSI in patients with non-mosaic Klinefelter syndrome: a comparative study. Reprod Biomed Online.

[20] Madureira C, Cunha M, Sousa M, Neto A, Pinho M, Viana P (2014). Treatment by testicular sperm extraction and intracytoplasmic sperm injection of 65 azoospermic patients with non-mosaic Klinefelter syndrome with birth of 17 healthy children. Andrology.

[21] Nahata L, Yu RN, Paltiel HJ, Chow JS, Logvinenko T, Rosoklija I (2016). Sperm retrieval in adolescents and young adults with klinefelter syndrome: a prospective, pilot study. J Pediatr.

[22] Emre Bakircioglu M, Erden HF, Kaplancan T, Ciray N, Bener F, Bahceci M (2006). Aging may adversely affect testicular sperm recovery in patients with Klinefelter syndrome. Urology.

[23] Bakircioglu ME, Ulug U, Erden HF, Tosun S, Bayram A, Ciray N (2011). Klinefelter syndrome: does it confer a bad prognosis in treatment of nonobstructive azoospermia?. Fertil Steril.

[24] Sabbaghian M, Modarresi T, Hosseinifar H, Hosseini J, Farrahi F, Dadkhah F (2014). Comparison of sperm retrieval and intracytoplasmic sperm injection outcome in patients with and without Klinefelter syndrome. Urology.

[25] Rogol AD, Skakkebaek NE (2016). Sperm retrieval in adolescent males with Klinefelter syndrome: medical and ethical issues. Transl Pediatr.

[26] Okada H, Goda K, Yamamoto Y, Sofikitis N, Miyagawa I, Mio Y (2005). Age as a limiting factor for successful sperm retrieval in patients with nonmosaic Klinefelter's syndrome. Fertil Steril.

[27] Bazán JL, Bolfarine H, Branco MD (2010). A framework for skew-probit links in binary regression. Communications in Statistics-Theory and Methods.

[28] Czado C, Fahrmeir L, Francis B, Gilchrist R, Tutz G (1992). On link selection in generalized linear models. Advances in GLIM and statistical modelling.New York: Springer-Verlag Inc.

